# Une cause rare de compression médullaire : l'hématome épidural rachidien spontané

**DOI:** 10.11604/pamj.2017.26.96.8174

**Published:** 2017-02-24

**Authors:** Abderrazzak El Saqui, Mohamed Aggouri

**Affiliations:** 1 Service Neurochirurgie, CHU Hassan II, Fès, Maroc

**Keywords:** Compression médullaire, l´hématome épidural, l´hémostase, Medullary compression, epidural hematoma, hemostasis

## Image en médecine

Patiente âgée de 26 ans, sans antécedents, admise en urgence pour prise en charge d'une lourdeur des 2 membres inférieurs. La symptomatologie a été débuté trois jours avant son admission par l'apparition brutale de rachialgies cervico-thoraciques, d'emblée intenses résistantes aux antalgiques habituels, suivies d'une lourdeur des deux membres inférieurs associées à des troubles génito-sphinctériens à type de rétention aigue d'urines et une constipation. L'interrogatoire n'a pas trouvé la notion de traumatisme ni de prise d'anticoagulants ou d'autre antécédent médico-chirurgical notable. L'examen clinique a objectivé une patiente consciente, apyrétique, ayant une paraplégie flasque grade A de Frankel avec une anesthésie à niveau supérieur mamelonnaire. Une IRM médullaire urgente a objectivé un hématome extradural (épidural) rachidien postérieur étendu de la septième vertèbre cervicale C7 à la deuxième vertèbre dorsale D2. Le Bilan biologique notamment le bilan de la crase sanguine était normal. La patiente fut opérée en urgence au travers une incision médiane postérieure avec réalisation d'une laminectomie étendue de la sixième vertèbre cervicale jusqu'à la deuxième vertèbre dorsale. L'hématome épidural a été évacué, l'hémostase faite et des prélèvements pour étude anatomopathologique ont été faits. L'étude histologique n'a pas retrouvé d'étiologie sous-jacente. L'évolution postopératoire a été marquée après un recul de six mois par une récupération complète du déficit moteur. L'hématome épidural rachidien spontané est une affection rare et grave. C'est une urgence neurochirurgicale, diagnostique et thérapeutique dont la rapidité de la prise en charge conditionne le pronostic. Le diagnostic est devenu aisé surtout avec l'avènement de l'IRM. Les facteurs de gravité sont l'installation brutale et rapide du déficit neurologique, la présence d'un déficit neurologique complet et la localisation thoracique de l'hématome. Une récupération neurologique précoce, un déficit incomplet ou essentiellement moteur, une localisation cervicale ou lombo-sacrée sont des facteurs d'une meilleure récupération.

**Figure 1:**
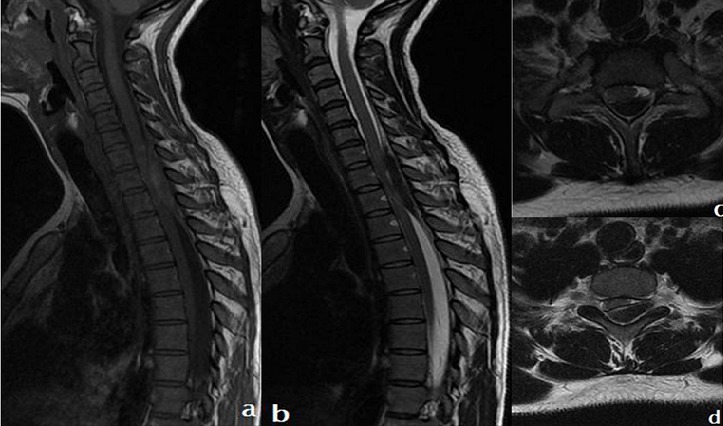
IRM du rachis cervico-dorsal en séquences T1 (a,c) et T2 (b,d) en coupes sagittales et axiales montrant une collection épidurale postérieure cervico-dorsale en hyposignal en T1 et T2, étendue en hauteur de C7 à D3 déterminant une compression de la moelle contre le mur postérieur du corps vertébral avec hypersignal de souffrance médullaire: Hématome épidural postérieur cervicodorsal à la phase aigue

